# Methodological Variation in Economic Evaluations Conducted in Low- and Middle-Income Countries: Information for Reference Case Development

**DOI:** 10.1371/journal.pone.0123853

**Published:** 2015-05-07

**Authors:** Benjarin Santatiwongchai, Varit Chantarastapornchit, Thomas Wilkinson, Kittiphong Thiboonboon, Waranya Rattanavipapong, Damian G Walker, Kalipso Chalkidou, Yot Teerawattananon

**Affiliations:** 1 Health Intervention and Technology Assessment Program, Nonthaburi, Thailand; 2 NICE International, National Institute for Health and Care Excellence, London, United Kingdom; 3 Global Health Program, Bill and Melinda Gates Foundation, Seattle, Washington, United States of America; Mahidol-Oxford Tropical Medicine Research Unit, THAILAND

## Abstract

Information generated from economic evaluation is increasingly being used to inform health resource allocation decisions globally, including in low- and middle- income countries. However, a crucial consideration for users of the information at a policy level, e.g. funding agencies, is whether the studies are comparable, provide sufficient detail to inform policy decision making, and incorporate inputs from data sources that are reliable and relevant to the context. This review was conducted to inform a methodological standardisation workstream at the Bill and Melinda Gates Foundation (BMGF) and assesses BMGF-funded cost-per-DALY economic evaluations in four programme areas (malaria, tuberculosis, HIV/AIDS and vaccines) in terms of variation in methodology, use of evidence, and quality of reporting. The findings suggest that there is room for improvement in the three areas of assessment, and support the case for the introduction of a standardised methodology or reference case by the BMGF. The findings are also instructive for all institutions that fund economic evaluations in LMICs and who have a desire to improve the ability of economic evaluations to inform resource allocation decisions.

## Introduction

Increasing demand for health services together with the accelerating developments in health technology place an ever-increasing strain on limited health resources. Health economic evaluation measures resources used against the outcomes of alternative policy options [1]. The ultimate aim of health economic evaluation is to improve resource allocation decisions by addressing efficiency in healthcare. Over the past decade, this method has gained increasing attention from decision makers in both resource-rich and resource-poor countries as well as among global health funders [2–4].

Established in 2000, an aim of the Bill & Melinda Gates Foundation (BMGF) is to enhance healthcare through supporting technology development in areas beset by particular health problems, including neglected tropical diseases and vaccine preventable diseases. Since 2004, BMGF has provided cumulative funding in excess of US$200 million for cost-effectiveness analysis and related activities around the world.

To maximise the benefit of economic evaluation information to health policy decisions, it is essential that the studies are comparable both within and across health problems as well as properly performed and reported to effectively assist the health investment decisions that could subsequently have a large impact on the health of target populations. Limited methodological quality is reported to be a significant barrier to the effective use of economic evaluation information [5–7]. This is of particular concern in low- and middle- income countries (LMICs) where research capacity in this field and reliable data sources are insufficient [7,8], and there are few methodological guidelines for performing locally-relevant economic evaluation [9]. As a result, BMGF, being a major funder of this type of research, aims to pioneer the development of a reference case for conducting health economic evaluations in developing countries to be referred to not only by its grantees but also by researchers who receive financial support from other funders.

Prior to this review, there was substantial uncertainty regarding the number, quality, methodology and focus of BMGF-funded economic evaluations as there was no repository or centralised collation mechanism. A key element of the reference case development and the aim of this review was to present a snapshot of the current status of BMGF-funded economic evaluations in focus programme areas.

## Methods

### Scope

The review included published economic evaluations undertaken in LMICs from 2000 onwards in four focus programme areas for BMGF [10] (vaccine, tuberculosis, HIV/AIDS, and malaria). The initial review identified all types of economic evaluation (cost-minimization; cost-effectiveness analysis; cost-utility analysis; cost-benefit analysis [1]) to provide an indicator of the proportion of economic evaluations that are supported by BMGF. The in-depth analyses included variation in methodology, quality of reporting, and quality of evidence used and was limited to economic evaluations that used the cost per disability adjusted life year (DALY)-averted outcome measure and were funded by BMGF. The review scope was limited to cost-per-DALY studies as i) BMGF sought consistency with the outcome measure of existing programmes that it funds such as the Global Burden of Disease initiative; ii) a cost-utility-study focus was required as the intention of the reference case was to improve the ability of economic evaluation to inform resource allocation decisions in terms of both allocative and technical efficiency, iii) a single study type facilitated meaningful inference from within the time and resources available for the analysis.

### Search Strategy

The search aimed initially to identify all economic evaluations in LMIC settings relating to the four programme areas to explore the number of existing studies during the time that BMGF had been established and examine the proportion of studies funded by BMGF. These were then narrowed down further to include only BGMF-funded studies that used cost-per DALY as their most aggregated measure of outcome for the in-depth analysis. To identify these economic evaluations, systematic reviews of this type of studies were firstly retrieved. Since BMGF was established in 2000, the period for the search of published systematic reviews in MEDLINE and Centre for Reviews and Dissemination databases (CRD) were limited to 2000 to May 2013. Individual economic evaluations were then identified manually through the citations in the relevant systematic reviews. An economic evaluation study was considered relevant if it was a full economic evaluation, i.e. studies that contain comparison of both cost and health outcomes of at least two alternatives [1]; conducted in LMIC settings and published from 2000 onwards. The studies that met criteria were then investigated for their funding sources to identify those which were funded by BMGF.

The search strategies used relevant terms of economic evaluation, including "economic evaluation", "cost-effectiveness", "cost-utility", "cost-benefit", "economic evaluations", "cost effectiveness", "cost utility", and "cost benefit" and the terms of intervention of interest, including vaccine, HIV/AIDS, tuberculosis/TB, and malaria, and the filter ‘systematic review’ was applied.

### Analytical Framework

All economic evaluations which were conducted in LMIC settings and published from 2000 onwards were analysed for their funding source and outcome measures. Most aggregate outcome measure, i.e. the measure that capture the most aspects among the measure used in that study, were considered. Therefore, if a study adopted both death averted and DALY, it would be categorised as using DALY as the aggregate measure.

The analytical frameworks developed by Walker and Fox-Rushby [11] and Teerawattananon et al. [12] for identifying method variations in economic evaluations conducted in LMICs were used on the included BMGF-funded cost-per-DALY studies. The analyses consisted of two parts. First, the manner of reporting, i.e. whether researchers reported adequate details, was explored using a number of variables adapted from Consolidated Health Economic Evaluation Reporting Standards (CHEERS) statement [13]. This included i) describing intervention and comparator(s) and the reason of choosing the comparator(s) ii) reporting characteristics of target populations iii) describing the perspective adopted iv) reporting horizon used v) reporting that discounting of costs and outcomes was done where relevant vi) informing unit,i.e. currency, and price date of cost data applied to the study and, if borrowed from other sources, how the cost data was converted to the study currency and price date vii) describing all key model parameter if a model was used viii) reporting Incremental cost-effectiveness ratios (ICERs) ix) discussing generalizability/transferability of the findings x) discussing equity consideration and affordability x) informing role of funders in the design and conduct of the study and xi) describing conflict of interest. Further examination was done to explore variation in methodology for certain variables, i.e. study perspective, analytical approach, uncertainty analysis, methods for currency conversion, methods for DALY calculation, and the threshold used. Second, quality of evidence used was evaluated using an adapted framework for hierarchy of evidence [14] (Table 1). Since the in-depth analysis was done only for cost-per-DALY studies, the hierarchy of the evidence of utilities such as those recommended by Cooper et al. [14] was not considered.

**Table 1:** Hierarchies for data sources, reproduced from Cooper et al., 2005 [14].

Rank	Data components
	**Clinical effect sizes/adverse events and complications**
1+	Meta-analysis of RCTs with direct comparison between comparator therapies, measuring final outcomes
1	Single RCT with direct comparison between comparator therapies, measuring final outcomes
2+	Meta-analysis of RCTs with direct comparison between comparator therapies, measuring surrogate outcomes. Meta-analysis of placebo-controlled RCTs with similar trial populations, measuring the final outcomes for each individual therapy
2	Single RCT with direct comparison between comparator therapies, measuring the surrogate outcomesSingle placebo-controlled RCTs with similar trial populations, measuring the final outcomes for each individual therapy
3+	Meta-analysis of placebo-controlled RCTs with similar trial populations, measuring the surrogate outcomes
3	Single placebo-controlled RCTs with similar trial populations, measuring the surrogate outcomes for each individual therapy
4	Case control or cohort studies
5	Non-analytic studies (e.g. case reports, case series)
6	Expert opinion
9	Not clearly stated
	**Baseline clinical data (if applicable)**
1	Case series or analyses of reliable administrative databases specifically conducted for the study covering patients solely from the jurisdiction of interest
2	Recent case series or analyses of reliable administrative databases covering patients solely from the jurisdiction of interest
3	Recent case series or analyses of reliable administrative databases covering patients solely from another jurisdiction
4	Old case series or analyses of reliable administrative databases. Estimates from RCTs
5	Estimates from previously published economic analyses:
6	Expert opinion
9	Not clearly stated
	**Costs**
1	Cost calculations based on reliable databases or data sources conducted for specific study: same jurisdiction
2	Recently published cost calculations based on reliable databases or data course: same jurisdiction
3	Data source not known: same jurisdiction
4	Using charge (price) rather than cost when societal perspective was adopted
5	Recently published cost calculations based on reliable databases or data sources: different jurisdiction
6	Data source not known: different jurisdiction
9	Not clearly stated
	**RCT** = randomised control trial.

## Results

### General profiles of the review

**Fig 1:**
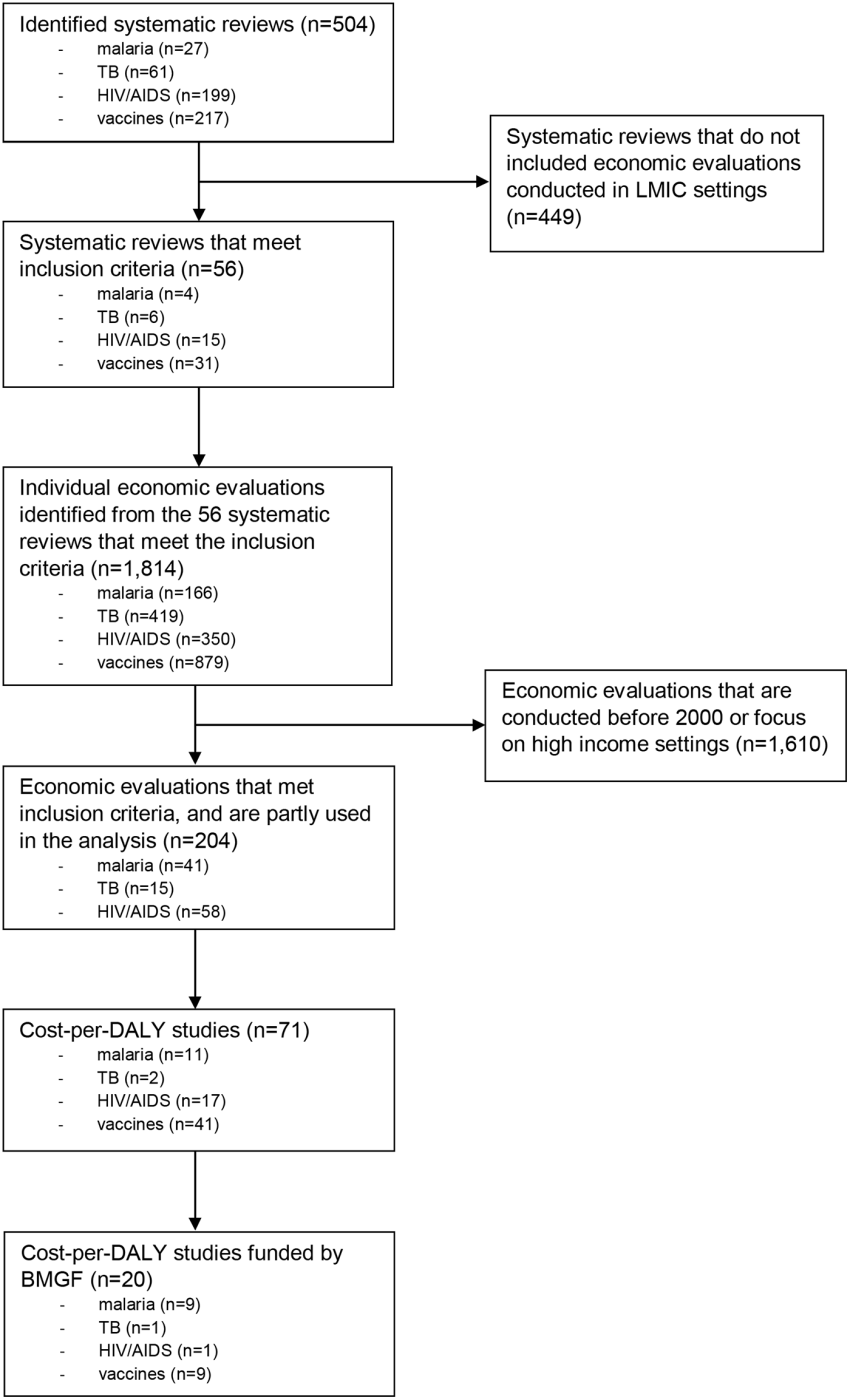
Flow of study selection.

Our study identified 56 eligible published systematic reviews of economic evaluations (Fig 1), of which the search period of those identified systematic reviews did not include economic evaluations published in 2012 and 2013. None of the systematic reviews except one (for Tanzania [15]) focused on a particular setting but instead included published literature conducted in low-, middle-, and high-income countries. From those systematic reviews, 204 economic evaluation articles were found to meet the inclusion criteria. The majority of the economic evaluations focused on vaccines (90 studies), followed by HIV/AIDS (58 studies), malaria (41 studies), and tuberculosis (TB) (15 studies). In total, there were 47 economic evaluations, including 20 cost-per-DALY studies (see S1 List of included cost-per-DALY studies), funded by the BMGF (23% of 204 studies) (Table 2).

**Table 2:** Number of identified economic evaluations by type of funder, country income level of setting where the economic evaluation was conducted, and area of interest.

Programme area	SR abstracts identified	SR matching inclusion criteria	EEs in included SRs	EEs matching inclusion criteria	Included EE funded by BMGF
Malaria	27 (5.4%)	4 (7.1%)	166 (9.1%)	41 (20.1%)	15 (31.9%)
*using DALY-averted outcome measure*				*17 (23*.*9%)*	*9 (45*.*0%)*
TB	61 (12.1%)	6 (10.7%)	419 (22.8%)	15 (7.4%)	1 (2.1%)
*using DALY-averted outcome measure*				*2 (2*.*8%)*	*1 (5*.*0%)*
HIV/AIDS	199 (39.5%)	15 (26.8%)	350 (19.1%)	58 (28.4%)	5 (10.6%)
*using DALY-averted outcome measure*				*11 (15*.*5%)*	*1 (5*.*0%)*
Vaccines	217 (43.1%)	31 (55.4%)	899 (49.0%)	90 (44.1%)	26 (55.3%)
*using DALY-averted outcome measure*				*41(57*.*7%)*	*9 (45*.*0%)*
**Total**	**504**	**56**	**1,834**	**204**	**47**
***using DALY-averted outcome measure***				***71***	***20***

SR: systematic review; EE: economic evaluation; BMGF: Bill and Melinda Gates Foundation; DALY: Disability-Adjusted Life Year; TB: tuberculosis

**Fig 2:**
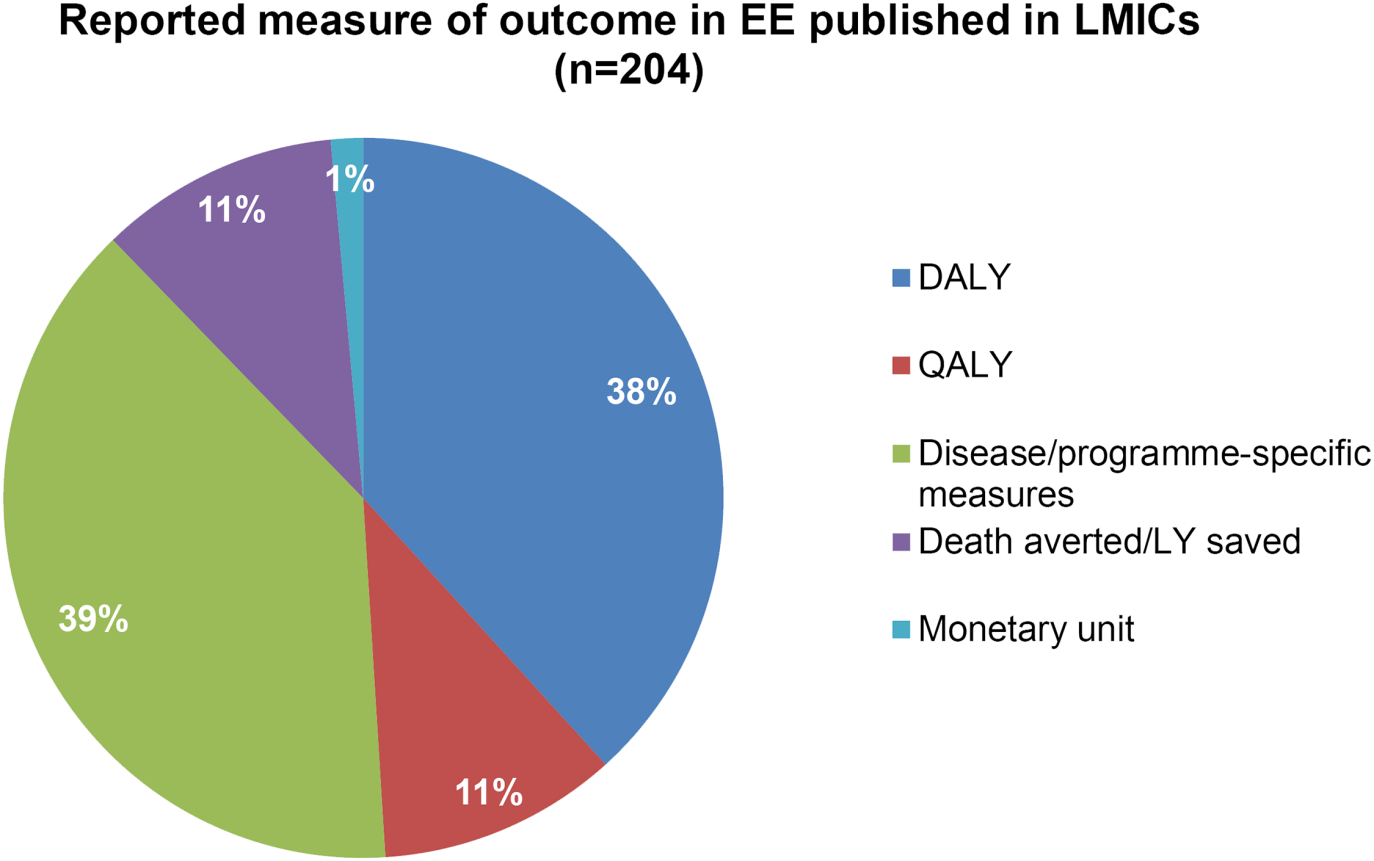
Most aggregated outcome reported in EEs published in LMICs, either funded by BMGF or not (n = 204). DALY: Disability-Adjusted Life Year; QALY: Quality-Adjusted Life Year; LY: life year.

Although the majority of studies were funded by non-BMGF organisations, BMGF was most often cited as the funding body compared to any other individual organization, except in area of TB, of which only one study was supported by the foundation. Disease/programme-specific measures such as infection averted were the most common aggregated outcome measures, reported in 39% of studies. Death averted or life year saved were the outcome measured in 11% of studies, while DALY averted and QALY were the most aggregated outcome measures for 38% and 11% of studies respectively. Only two studies (1%) used monetary benefit (cost-benefit analyses) as an outcome measure. (Fig 2).

**Fig 3:**
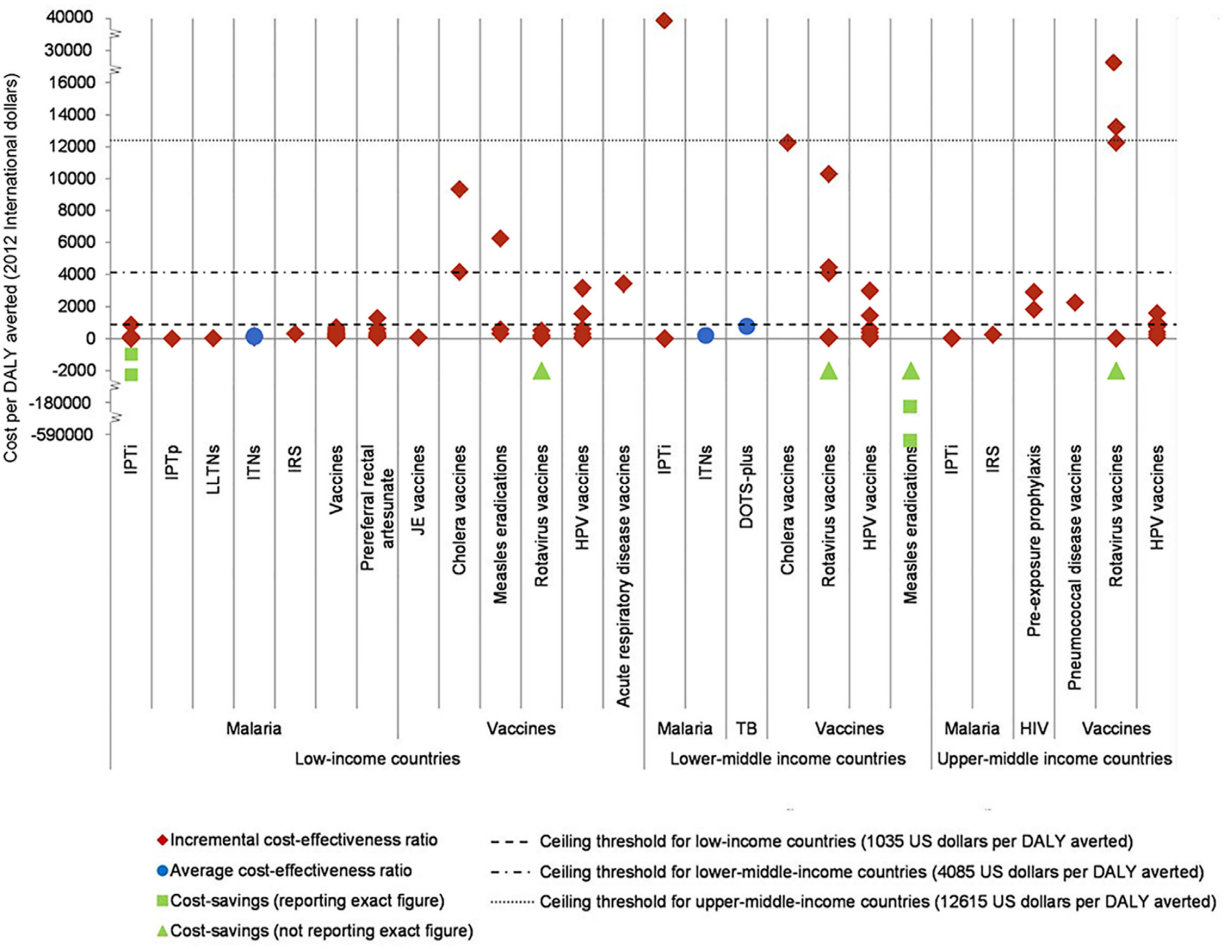
Cost-effectiveness league chart showing ICERs of interventions being evaluated in identified BMGF-funded cost-per-DALY studies (n = 20). IPTi: Intermittent preventive treatment for infants, IPTp: Intermittent preventive treatment for pregnant women, LLTNs: Long-lasting treated nets, ITNs: Insecticide treated nets, IRS: Indoor residual spray, JE: Japanese encephalitis, HPV: Human papilloma virus, DOTS: Directly observed treatment, short course. Source of consumer price index and purchasing power parity: IMF World economic outlook database.

ICERs derived from each BMGF-funded cost-per-DALY study illustrate that almost all of the interventions in the areas of malaria, TB and HIV/AIDS represented good value for money (as defined by the World Health Organization) as the reported ICERs were below a ceiling threshold equal to Gross National Income per capita (1035 US dollars for low-income countries using World Bank classifications) [16] (Fig 3). It is noteworthy that in the case of vaccines, different settings yielded fairly different ICERs, which may be due to several factors including variation in epidemiology such as disease incidence/prevalence [17]. However, this can also be a consequence of the difference in methodological approaches rather than the true differences in effectiveness or costs of the evaluated interventions.

### Manner of reporting and variation in methodology of cost-per-DALY studies funded by the BMGF

**Fig 4:**
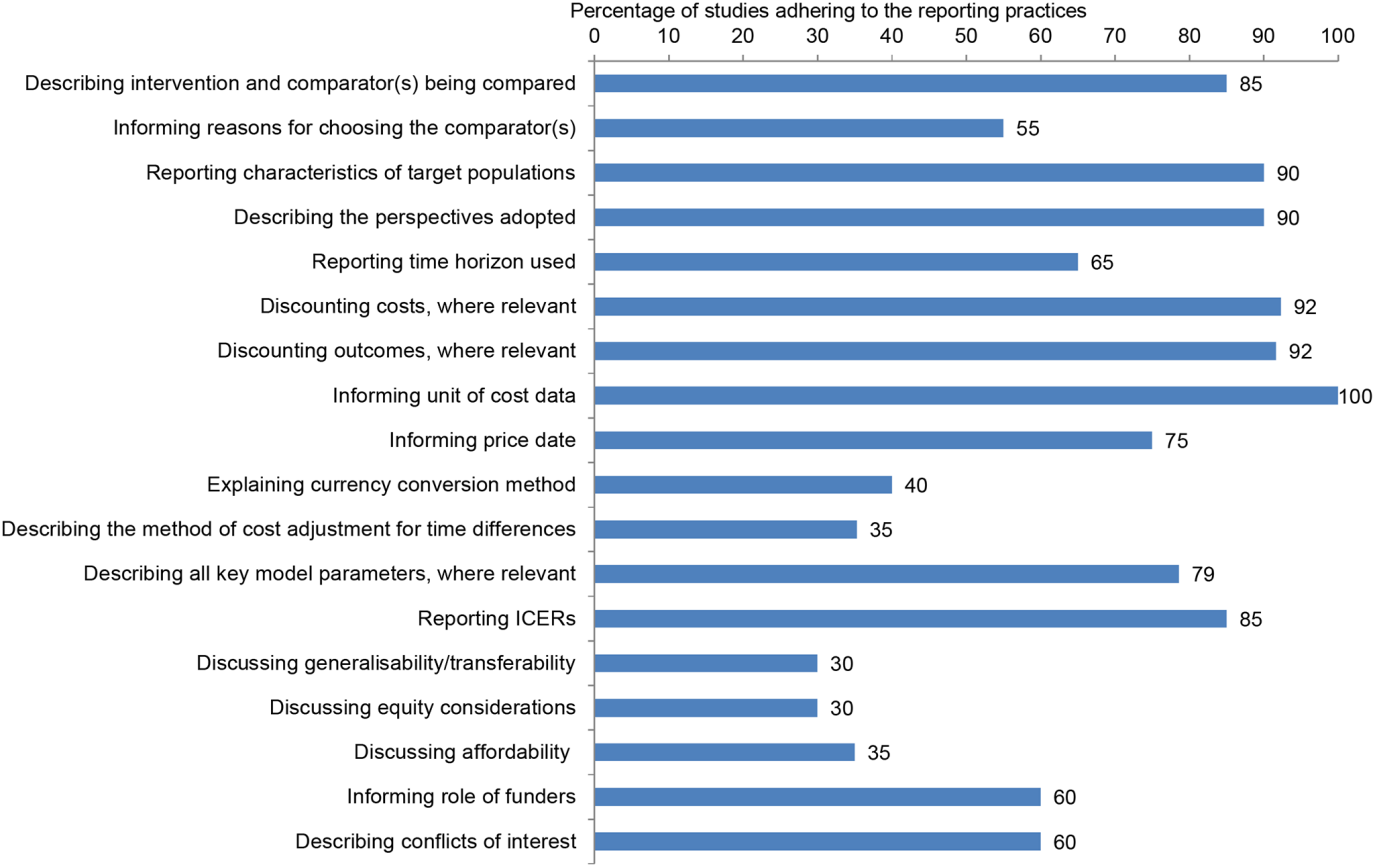
Percentage of BMGF-funded cost-per-DALY studies adhering to good practices for reporting health economic evaluations adapted from CHEERS statement [13] (n = 20).

The percentage of cost-per-DALY studies funded by the BMGF adhering to a set of reporting requirements is shown in Fig 4. Generalisability/transferability and equity considerations were the attributes most often neglected in the studies, followed by affordability, price date, method of cost adjustment for time difference between price date in source for cost data and price date in the study and method of currency conversion.

Further analyses suggested that there was significant heterogeneity in methodology used in the cost-per-DALY studies funded by the BMGF. Most studies (12 out of 20 studies) were conducted using a societal perspective, followed by healthcare provider (5 studies [18–22]) and health system (1 study [23]) viewpoints, and two studies did not clearly state the perspective used. Regarding the analytical approach, 13 studies were model-based, of which four studies [20,22,24,25] constructed a decision tree; two studies [26,27] adopted a Markov model; two studies [28,29] applied a dynamic model; one study [21] used a mathematical model; four studies [30–33] did not specify the type of model used. Considering uncertainty analysis, most studies performed univariate or multivariate sensitivity analysis while only two studies [20,23] conducted a probabilistic sensitivity analysis. In some studies [23,25–27,32], a threshold analysis was also carried out along with the sensitivity analyses. More than half of the studies (12 studies) did not describe the method used for converting currencies even though they borrowed cost data from sources outside their study settings. Exchange rates were more frequently used (5 studies [18,19,25,31,34]) rather than purchasing power parity (PPP) (3 studies [17,24,32]) for converting foreign cost data to the local currency of the study setting.

Most studies did not follow the specific methodological recommendations of the Global Burden of Disease Project for the calculation of DALYs [35]. Only the study by Mbonye et al. [36] adhered to all three major methodological specifications, namely using a standard life table, applying age-weighting, and performing discounting for future DALYs. Four studies [21–23,25] used age-weighting and discounting but not standard life table. Thirteen out of the 20 studies discounted future DALYs but did not apply age-weighting and standard life table. Two studies [34,37] did not clearly state whether any of the recommendations were applied. When discounting was relevant and performed, 3% was the rate used, not only for DALYs but also for cost data. Eleven out of 20 studies described the choice of study comparator(s). Comparators representing current or first-line practice were most commonly adopted (6 studies). Approximately half of the studies referred to a ceiling threshold of 1 to 3 times of capita GDP per DALY gained [38] as the decision rule for determining if a particular technology was good value for money. Seven out of 20 economic evaluations made a recommendation for adopting the technology based on the above decision rule. Lastly, twelve studies clearly informed the role of funders in the study design and conduct.

### Quality of evidence used

**Fig 5:**
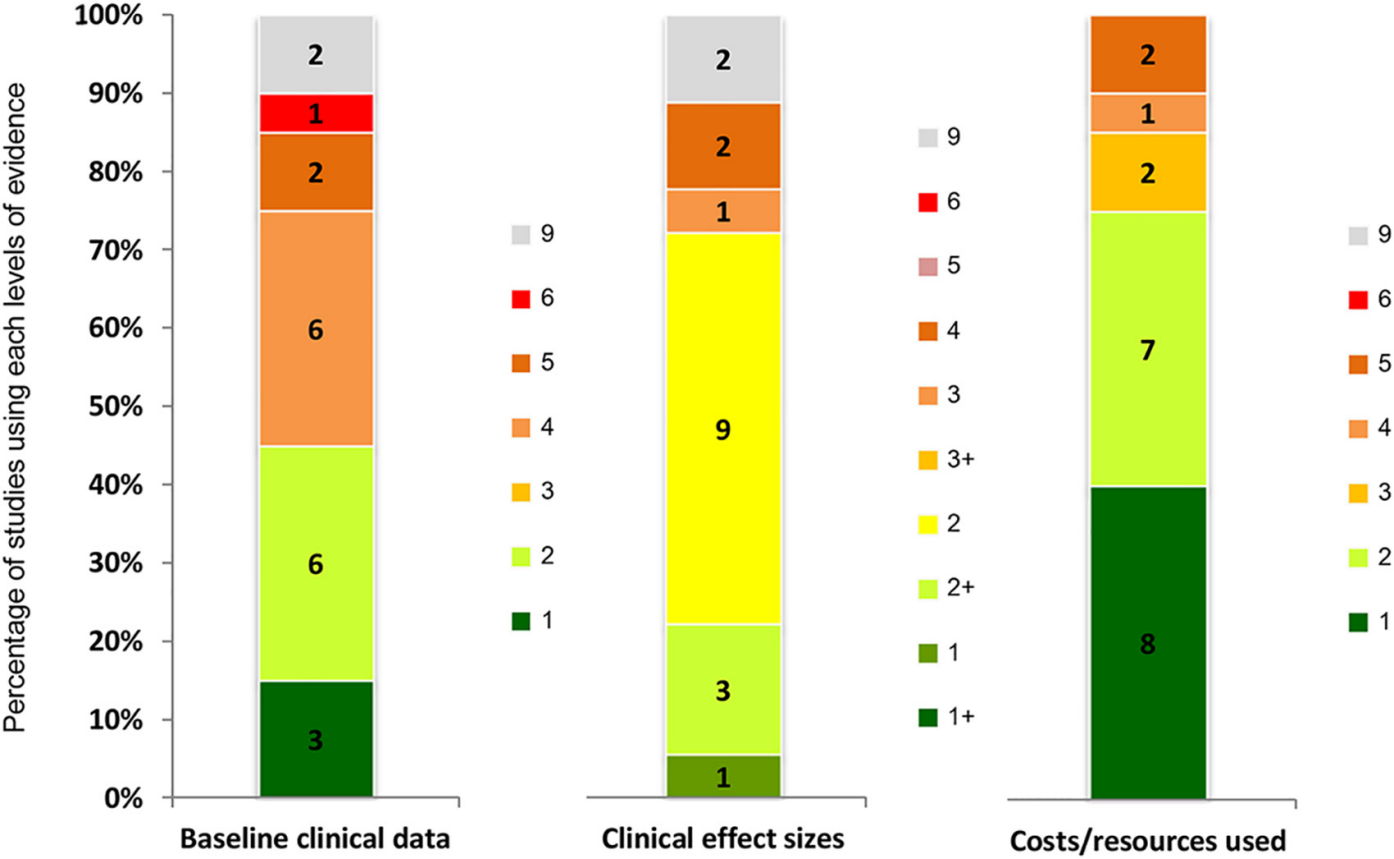
Ranks of evidence used in the included BMGF-funded cost-per-DALY studies (n = 20). Full details of hierarchy of evidence were provided in Table 1 [14].

Studies generally employed a higher quality of evidence for cost and resource parameters compared to other parameters, with the majority of studies estimating costs based on reliable administrative databases or data sources conducted for specific studies in the same jurisdiction. Baseline clinical data were often derived from relatively low data quality sources, e.g. case series, administrative databases. Similarly, the clinical effect sizes were mostly retrieved from a single RCT (Fig 5).

## Discussion

There is an increasing trend of conducting economic evaluations for the purpose of informing resource allocation decision-making in LMICs [39,40], driven largely by increased investment in this kind of policy research by major global health players such as BMGF, GAVI alliance, and the World Health Organization. Although we believe that economic evaluation is a useful priority setting tool, it is far from perfect, especially in a situation where there are no uniform methodological approaches and reporting standards due to numerous methodological controversies and variations, as well as the possibility of biases being introduced in many ways and at various stages of the analysis [41,42]. Also, poor reporting quality is likely to restrict the usefulness of economic assessment in policy decision-making [12,13]. This review summarises key issues arising from the review of cost-per-DALY studies published in international journals in selected areas.

The review indicates that

- Methodological variation across economic evaluations is significant in almost every component except for the discount rate used.
- Societal and health care provider’s viewpoints are among the most popular study perspectives used. Because household expenditure can be substantial, the use of these different perspectives can easily generate different conclusions even for studies in the same setting, focusing on the same intervention. Difference in perspective adopted among the studies reviewed may be due to the difference in primary audience of the study results, but if the studies aim to inform the same audience, the perspective used should be consistent.
- There is considerable disparity in the costing methods used. A majority of studies do not offer sufficient information about currency conversion and method of cost adjustment for time differences. For those giving adequate information, the exchange rate is often used to convert unit costs borrowed from other settings with more reliable data sources (often resource-rich countries).
- Despite the fact that purchasing power can better reflect opportunity cost of using resources across different settings, PPP was used in only a few studies. This may be explained by the fact that exchange rates are better understood by not only decision makers but also the general public.
- The poor adherence to the three methodological specifications for DALY estimation which were recommended during the period the studies were conducted raises concern—the eight possible approaches to DALY calculation result in difficulties when making cross-study comparisons. However, it is noteworthy that the latest recommendation from the Global Burden of Disease research program [43] has changed, omitting age-weighting and discounting.
- Only a few (2 out of 20) economic evaluations employed probabilistic sensitivity analysis even though it is widely recommended in resource-rich settings as the most comprehensive method of dealing with various forms of uncertainty in economic evaluation [44].
- The findings of this review are consistent with previous reviews which found better quality of cost and resource data were used for economic evaluations in resource-limited settings compared to baseline clinical data and clinical effect sizes [12]. This may be due to the lack of reliable administrative databases or existing costing studies prompting researchers to conduct primary cost studies.
- Generalisability/transferability of results and equity implication of evaluated interventions are only discussed in less than one-third of all reviewed studies.
- Perhaps the most surprising result is that only 35 percent of the studies discussed the affordability of the interventions being assessed, which is particularly poignant given that these studies were conducted in resource-limited settings.

### Implications for the way research is conducted

This review highlights the fact that serious attention needs to be given to the quality of reporting and consistency of the analyses, especially with regard to the following points:

- It is important to adhere to good practice criteria for reporting economic evaluations including providing reasons for choosing the comparator, describing the method of performing currency conversion, and the method of adjustment for time difference between date of cost collection and the analysis.
- As generalisability/transferability of results and equity implications of evaluated interventions are important issues in order to make use of the research finding, they should be discussed.
- Since information on affordability of the evaluated technology is an important input for policy decision-making, it should be emphasised in the discussion.
- The roles of funders and potential conflicts of interest should also be better addressed in future studies.
- There is a need for uniform methodological specifications and reporting standards for conducting health economic evaluations in LMICs for the purpose of improving the quality and reducing the disparity in the methods and reporting used for future studies.

We hope our recommendations will help ensure standards that facilitate value-for-money comparisons of health interventions being considered for introduction in resource-limited settings. Without any standardisation of methods, the differences in a cost-effectiveness ratio may arise from differences in study methodology rather than reflecting true differences between the interventions being evaluated in a given setting.

This review provides an indicator of the variation in methodological approach, use of evidence and quality of reporting in cost-per DALY, BMGF-funded economic evaluations in four programme areas. The findings contribute to the multi-stakeholder and to the production of a BMGF reference case, not only by identifying key methodological areas that should be addressed within the reference case, but also by providing an indication of priority for methodological research to support the use of a reference case by BMGF-grantees.

### Implications for funders and policy makers

This review serves not only to inform the development of a reference case for BMGF, but also to provide insights for local governments, and global, regional and local development partners who wish to make evidence-informed decisions to recognize potential problems in terms of quality and comparability of studies if there is no standard methodological guideline for conducting economic evaluations. Although there are instances of high-quality economic evaluations in LMIC settings, their variability in quality and comparability limits their routine use as a source of evidence for policy formation. This indicates that the reference case would not only be of benefit to BMGF, but also to the wider donor community and local decision makers if it was adopted more widely to enable the improvement of the quality and usefulness of evidence produced by all economic evaluations in LMIC contexts.

### Limitations

It is important to point out the limitations of this review. The major limitation was due to the 2-stage search method, i.e. published systematic reviews of health economic evaluations were firstly identified, and then full economic evaluation papers recognised from the citations of those systematic reviews were retrieved. This may result in the omission of individual economic evaluations excluded from the identified systematic reviews. Moreover, this review considers systematic reviews that were published in English only. Thus, the search excluded conference proceedings, master and doctoral theses as well as ‘grey literature’ such as government reports as well as publications in other languages. However, the results tended to remain valid regardless of the limitations due to the search strategy. As the included studies were published in leading reputable international journals with relatively strong review process, including unpublished material and grey literature works would most likely have resulted in greater variation of methods applied as well as a lower quality of reporting and evidence used. The scope of the quality assessment section of the review limited inclusion of BMGF-funded studies. While this was appropriate to inform a reference case for use by BMGF, this does limit unqualified generalisation of the findings to all economic evaluation regardless of funding source. However, as BMGF was shown to be the largest funder of economic evaluation in LMIC in the included disease areas, we consider that the findings provide a useful indication of the quality of economic evaluations funded by other sources. Moreover, the review focuses on malaria, TB, HIV/AIDS, and vaccines, all of which have received strong support from major global health donors, including Global Fund, the BMGF, and GAVI Alliance. In contrast, other neglected tropical diseases might not have had as many economic evaluation studies of similar quality as those four mentioned areas. Third, it would be of interest to assess the improvement of study quality and reporting over time. However, due to the relatively small number of studies that met our inclusion criteria (use of cost-per DALY as an outcome measure and BMGF funding) this was not possible.

## Supporting Information

S1 List List of included cost-per-DALY studies. (DOCX)
