# Supplementary material for: Methodological Variation in Economic Evaluations Conducted in Low- and Middle-Income Countries: Information for Reference Case Development
Source: PLoS One. 2015 May 7;10(5):e0123853. doi: 10.1371/journal.pone.0123853 (PMC4423853; doi:10.1371/journal.pone.0123853)
Supplement: S1 List — (DOCX) [file pone.0123853.s001.docx]

**S1: List of included cost-per-DALY studies**

1. Bishai D, Johns B, Lefevre A, Nair D (2010) Cost effectiveness of measles eradication. Johns Hopkins Bloomberg School of Public Health
2. Constenla DO (2008) Economic impact of pneumococcal conjugate vaccination in Brazil, Chile, and Uruguay. Rev Panam Salud Publica 24: 101-112.
3. Conteh L, Sicuri E, Manzi F, Hutton G, Obonyo B, et al. (2010) The cost-effectiveness of intermittent preventive treatment for malaria in infants in Sub-Saharan Africa. PLoS One 5: e10313.
4. Ding D, Kilgore PE, Clemens JD, Wei L, Zhi-Yi X (2003) Cost-effectiveness of routine immunization to control Japanese encephalitis in Shanghai, China. Bull World Health Organ 81: 334-342.
5. Goldie SJ, O'Shea M, Campos NG, Diaz M, Sweet S, et al. (2008) Health and economic outcomes of HPV 16,18 vaccination in 72 GAVI-eligible countries. Vaccine 26: 4080-4093.
6. Gomez GB, Borquez A, Caceres CF, Segura ER, Grant RM, et al. (2012) The potential impact of pre-exposure prophylaxis for HIV prevention among men who have sex with men and transwomen in Lima, Peru: a mathematical modelling study. PLoS Med 9: e1001323.
7. Hutton G, Schellenberg D, Tediosi F, Macete E, Kahigwa E, et al. (2009) Cost-effectiveness of malaria intermittent preventive treatment in infants (IPTi) in Mozambique and the United Republic of Tanzania. Bull World Health Organ 87: 123-129.
8. Jeuland M, Cook J, Poulos C, Clemens J, Whittington D (2009) Cost-effectiveness of new-generation oral cholera vaccines: a multisite analysis. Value Health 12: 899-908.
9. Kim SY, Goldie SJ, Salomon JA (2009) Cost-effectiveness of Rotavirus vaccination in Vietnam. BMC Public Health 9: 29.
10. Kim SY, Lee G, Goldie SJ (2010) Economic evaluation of pneumococcal conjugate vaccination in The Gambia. BMC Infect Dis 10: 260.
11. Kim SY, Sweet S, Slichter D, Goldie SJ (2010) Health and economic impact of rotavirus vaccination in GAVI-eligible countries. BMC Public Health 10: 253.
12. Mbonye AK, Hansen KS, Bygbjerg IC, Magnussen P (2008) Intermittent preventive treatment of malaria in pregnancy: the incremental cost-effectiveness of a new delivery system in Uganda. Trans R Soc Trop Med Hyg 102: 685-693.
13. Mueller DH, Wiseman V, Bakusa D, Morgah K, Dare A, et al. (2008) Cost-effectiveness analysis of insecticide-treated net distribution as part of the Togo Integrated Child Health Campaign. Malar J 7: 73.
14. Sicuri E, Bardaji A, Nhampossa T, Maixenchs M, Nhacolo A, et al. (2010) Cost-effectiveness of intermittent preventive treatment of malaria in pregnancy in southern Mozambique. PLoS One 5: e13407.
15. Tediosi F, Hutton G, Maire N, Smith TA, Ross A, et al. (2006) Predicting the cost-effectiveness of introducing a pre-erythrocytic malaria vaccine into the expanded program on immunization in Tanzania. Am J Trop Med Hyg 75: 131-143.
16. Tediosi F, Maire N, Penny M, Studer A, Smith TA (2009) Simulation of the cost-effectiveness of malaria vaccines. Malar J 8: 127.
17. Touch S, Suraratdecha C, Samnang C, Heng S, Gazley L, et al. (2010) A cost-effectiveness analysis of Japanese encephalitis vaccine in Cambodia. Vaccine 28: 4593-4599.
18. Tozan Y, Klein EY, Darley S, Panicker R, Laxminarayan R, et al. (2010) Prereferral rectal artesunate for treatment of severe childhood malaria: a cost-effectiveness analysis. Lancet 376: 1910-1915.
19. Tupasi TE, Gupta R, Quelapio MI, Orillaza RB, Mira NR, et al. (2006) Feasibility and cost-effectiveness of treating multidrug-resistant tuberculosis: a cohort study in the Philippines. PLoS Med 3: e352.
20. Yukich JO, Lengeler C, Tediosi F, Brown N, Mulligan JA, et al. (2008) Costs and consequences of large-scale vector control for malaria. Malar J 7: 258.
